# Supplementary material for: Splice-Junction-Based Mapping of Alternative Isoforms in the Human Proteome
Source: Cell Rep. Author manuscript; Available in PMC 2020 Jan 15. (PMC6961840; doi:10.1016/j.celrep.2019.11.026)
Supplement: 2 [file NIHMS1546469-supplement-2.zip › DF1/PXD006675/RightCoronaryArtery/RightCoronaryArtery_9_TRIM28_RSGAAHSAMCSLTAAAASAASGSPGPGEGSAGGEK.pdf]

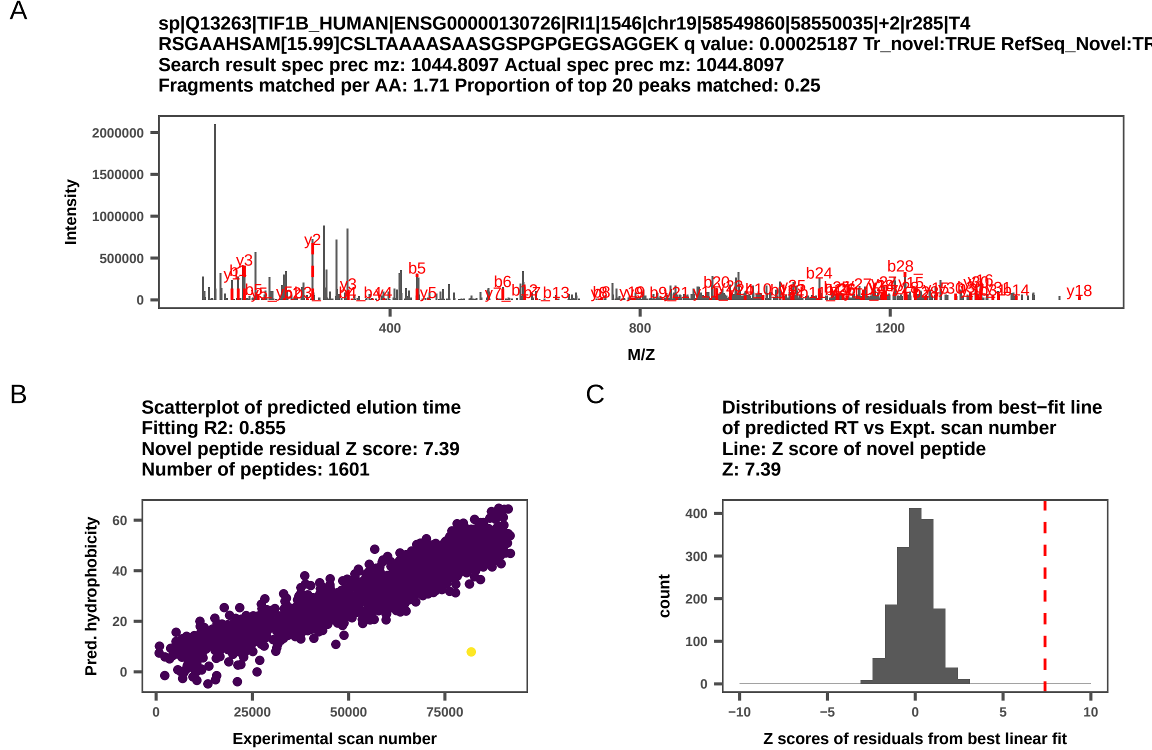
A
sp|Q13263|TIF1B_HUMAN|ENSG00000130726|RI1|1546|chr19|58549860|58550035|+2|r285|T4
RSGAAHSAM[15.99]CSLTAAAASAASGSPGPGEGSAGGEK q value: 0.00025187 Tr_novel:TRUE RefSeq_Novel:TR
Search result spec prec mz: 1044.8097 Actual spec prec mz: 1044.8097
Fragments matched per AA: 1.71 Proportion of top 20 peaks matched: 0.25
Intensity
2000000
1500000
1000000
500000
0
M/Z
400
800
1200
y2
y3
b5
b6
b24
b28_
y18
B
Scatterplot of predicted elution time
Fitting R2: 0.855
Novel peptide residual Z score: 7.39
Number of peptides: 1601
Pred. hydrophobicity
60
40
20
0
Experimental scan number
0
25000
50000
75000
C
Distributions of residuals from best−fit line
of predicted RT vs Expt. scan number
Line: Z score of novel peptide
Z: 7.39
count
400
300
200
100
0
Z scores of residuals from best linear fit
−10
−5
0
5
10
